# Comparison of open and laparoscopic outcomes of adult Type-I congenital choledochal cysts

**DOI:** 10.12669/pjms.39.6.7829

**Published:** 2023

**Authors:** Hui Lu, Wailin Hou

**Affiliations:** 1 Hui Lu Department of Wound Clinic Chenzhou First People’s Hospital, Chenzhou 423000, Hunan Province, P.R. China; 2 Wailin Hou Department of Hepatobiliary Surgery Chenzhou First People’s Hospital, Chenzhou 423000, Hunan Province, P.R. China

**Keywords:** Congenital choledochal cyst, Laparoscopy, Open surgery, Roux-en-Y hepaticojejunostomy

## Abstract

**Objective:**

To compare open and laparoscopic outcomes of adult Type-I congenital choledochal cysts.

**Methods:**

Clinical data of 78 adult patients with Type-I congenital choledochal cysts, who had undergone cyst resection and Roux-en-Y hepaticojejunostomy in Chenzhou First People’s Hospital from September 1, 2021 to August 31, 2022, were retrospectively analyzed. Patients who received open approach and Roux-en-Y hepaticojejunostomy constituted the open group (n=35,) and patients who received laparoscopic approach and Roux-en-Y hepaticojejunostomy were assigned into the laparoscopic group (n=43,). The intraoperative and postoperative conditions, relevant laboratory indicators, and the rate of complications were compared between the two groups.

**Results:**

Intraoperative blood loss, postoperative time to first flatus, diet recovery time, time to drainage tube removal, and length of hospitalization of the laparoscopic group were lower in the laparoscopic group compared to the open group (*P*<0.05). One day after the operation, serum amylase (SAMY) levels in both groups were significantly lower, while the levels of total bilirubin(TBIL), alanine aminotransferase(ALT), and C-reactive protein(CRP) were higher than before the operation. Postoperative SAMY level in the laparoscopic group was significantly higher, while the postoperative TBIL and CRP levels were significantly lower than those in the open group (*P<*0.05). The incidence of postoperative complications in the laparoscopy group (4.65%) was significantly lower than the open group (20.00%) (*P<*0.05).

**Conclusions:**

Laparoscopic cyst resection combined with Roux-en-Y hepaticojejunostomy is associated with lower extent of trauma, faster recovery, less inflammation, and fewer complications than open surgery in adult patients with Type-I congenital choledochal cysts.

## INTRODUCTION

Congenital choledochal cysts are biliary malformations that occur with an incidence of one in 100000-150000 live births in the Western population, and one in 1000 live births in the Asian population.^1^ The condition is characterized by spindle-shaped and cystic dilatation of the common bile duct, and, in some patients, intrahepatic bile duct dilatation, which can lead to abnormal confluence of bile and pancreatic ducts, poor drainage of bile and pancreatic juices, repeated biliary tract infections, biliary stones, biliary pancreatitis, and even cancer.^2^ Therefore, in adults, congenital choledochal cysts should be treated in a timely manner by surgically removing the cyst lesions and then reconstructing the biliary tract.

Roux-en-Y hepaticojejunostomy is a commonly used biliary reconstruction method. In the past, this procedure was mainly performed under direct vision during an open laparotomy. However, this surgical approach causes abundant tissue trauma and produces a strong stress response that increases the risk of complications and results in a long recovery time. 3,4

With the continuous development of laparoscopic surgery technologies, laparoscopic cyst resection combined with Roux-en-Y hepaticojejunostomy is becoming more popular for the treatment of congenital choledochal cysts. It has the advantages of minimally invasive surgery and rapid recovery. While there have been many studies on the clinical effect of laparoscopic cyst resection combined with Roux-en-Y hepaticojejunostomy in pediatric population, only few studies focused on adult patients. As laparoscopic cyst resection combined with biliary tract reconstruction is a complex operation,^5^ the studies on its potential effects in adults are scarce compared to laparotomy cyst resections combined with Roux-en-Y hepaticojejunostomy. Our study aimed to compare the two approaches for the treatment of adult Type-I congenital choledochal cysts to provide valuable information for surgeons.

## METHODS

Clinical records of 78 adult patients (32 men and 46 women) with Type-I congenital choledochal cysts treated in Chenzhou First People’s Hospital from September 2021 to September 2022 were retrospectively reviewed. The average age of the patients was 49.77±15.23 years. The patients were grouped based on the surgical method. Patients who received open laparotomy approach and Roux-en-Y hepaticojejunostomy comprised the open group (n=35,) and patients who received laparoscopic surgery and Roux-en-Y hepaticojejunostomy laparoscopic comprised the laparoscopy group (n=43,).

### Inclusion criteria:

- Patients with diagnosis of Type-I choledochal cyst confirmed by ultrasonography, CT, or other imaging examinations.^6^
- Patients older than 18 years.
- Patients with complete medical records.

### Exclusion criteria:

- Patients with prior biliary tract and upper abdomen operations.
- Patients with severe biliary malformations.
- Patients had severe complications such as diffuse peritonitis or cholestatic liver cirrhosis before operation.
- Patients with malignant lesions of the bile duct or pancreas.
- Patients who required conversion to laparotomy after laparoscopic surgery failure.

### Ethical approval:

The Medical Ethics Committee of Chenzhou First People’s Hospital approved this study (No. 2022011, Date: 2022-01-12).

All patients in the open group received open surgery (laparotomy) combined with Roux-en-Y hepaticojejunostomy, while the patients in the laparoscopic group underwent laparoscopic surgery combined with Roux-en-Y hepaticojejunostomy. For the open approach, after the anesthesia induction and routine disinfection for bowel operations, the surgeon performed a left side incision 15 to 20cm below the upper abdominal costal edge and then dissected the abdomen layer by layer to be able to explore the abdominal cavity. For the laparoscopic group, the surgeon punctured the skin 2cm below the umbilicus, and inserted a 100-mm laparoscope to explore the abdominal cavity and guide the sites of the next four puncture points. Four trocars (10-mm, 12-mm, 5-mm, and 5-mm in diameter) were respectively placed under the right xiphoid, the left clavicular midline flat umbilical cord, the right clavicular midline costal edge, and the right axillary front costal edge.

### Collection of basic clinical characteristics of patients and relevant indicators before and after the operations:

1) Data on operation times, intraoperative blood loss, postoperative times to flatus, diet recovery, times to drainage tube removal, and hospitalization lengths were collected. 2) The automatic biochemical analyzer was used to detect the levels of serum amylase (SAMY), total bilirubin (TBIL) and alanine aminotransferase (ALT), and immune turbidimetry was used to detect CRP levels. All measurements were taken on the day of the operation and a day after. 3) We also collected all the data on surgical complications from the computer record of the hospital, including occurrences of biliary fistulas, pancreatic fistulas, abdominal bleeding, abdominal infections, incision infections, and reflux cholangitis.

### Statistical analysis:

PSS22.0 was used to process the relevant data. Non-grade counting data were presented as numbers and percentages [n (%)] and tested using the *χ^2^* method. Normal distributed measurement data were presented as means and standard deviations and the significance of differences was tested using *t* tests, while the non-normal distributed measurement data were presented as median (interquartile range) and tested by Mann–Whitney U test. We considered differences with *P*<0.05 as statistically significant.

## RESULTS

A total of 78 patients (32 men and 46 women) met the inclusion criteria. There were no differences in the general data between the two groups (*p >*0.05) (Table-I).

**Table-I:** Comparison of general data of the two groups.

Group	n	Gender (men/women)	Age (years)	Diameter of cyst (cm)
Open-group	35	15/20	48.68±15.84	4.00(2.00-5.00)
Laparoscopy-group	43	17/26	50.65±14.84	4.00(3.00-5.00)
*χ^2^*/*t / Z*	-	0.088	-0.564	-0.466
*P*	-	0.767	0.574	0.641

Operation time was similar in both groups (*p >*0.05). The intraoperative blood loss, times to first flatus, diet recovery, and drainage tube removal, and the hospitalization lengths were lower in the laparoscopy group compared to the open group (*p <*0.05) (Table-II).

**Table-II:** Comparison of intraoperative and postoperative conditions between the two groups.

Group (n)	Operation time (minutes)	Intraoperative bleeding (mL)	Postoperative time to first flatus (day)	Diet recovery time (days)	Time to drainage tube removal (hours)	Length of hospitalization (days)
Open group (n=35)	160.83±26.53	54.00(48.00-62.00)	4.00(3.00-5.00)	5.00(3.00-5.00)	7.00(6.00-8.00)	11.00(10.00-12.00)
Laparoscopy group (n=43)	165.23±23.61	38.00(35.00-42.00)	3.00(2.00-3.00)	3.00(3.00-4.00)	5.00(5.00-6.00)	9.00(8.00-9.00)
*t/Z*	-0.775	-6.563	-6.123	-3.830	-5.650	-5.457
*P*	0.441	<0.001	<0.001	<0.001	<0.001	<0.001

We found similar levels of SAMY, TBIL, ALT, and CRP in both groups one day before the operation (*p* >0.05). One day after the operation, the levels of SAMY in both groups were significantly lower than those before the operations, while the levels of TBIL, ALT, and CRP were higher. In addition, postoperative SAMY level in the laparoscopy group was significantly higher than that in the open group, while the postoperative TBIL and CRP levels were significantly lower than those in the open group (*p <*0.05) (Table-III). The mean incidence of surgical complications in the laparoscopy group (4.65%) was significantly lower than the open group (20.00%) (p *<*0.05) (Table-IV).

**Table-III:** Comparison of laboratory indexes between the two groups (*χ̅*±*S*).

Group (*n*)	SAMY (U/L)		TBIL (µmol/L)		ALT (U/L)		CRP (mg/L)	

	One day before operation	One day after operation	One day before operation	One day after operation	One day before operation	One day after operation	One day before operation	One day after operation
Open-group (*n*=35)	86.00(82.00-95.00)	47.00(43.00-53.00)*	11.50(9.20-13.50)	17.00(14.80-19.40)*	30.22±3.82	39.62±4.23*	1.50(1.30-1.70)	43.50(42.30-46.80) *
Laparoscopy-group (*n*=43)	85.00(82.00-91.00)	51.00(48.00-57.00)*	10.50(8.90-12.60)	13.80(12.20-16.70)*	29.97±3.42	38.83±4.16*	1.50(1.30-1.80)	30.50(29.10-31.80)*
*t/Z*	-0.584	-3.746	-0.915	-4.467	0.307	0.828	-0.324	-7.562
*P*	0.559	<0.001	0.360	<0.001	0.760	0.410	0.746	<0.001

***Note:***

* comparison with preoperative value, p <0.05.

**Table-IV:** Comparison of surgical complications between the two groups [n (%)].

Group	n	Surgical complications						Total

		Biliary fistula	Pancreatic fistula	Celiac hemorrhage	Abdominal infection	Incision infection	Refluxing cholangitis	
Open-group	35	2 (5.71)	1 (2.86)	1 (2.86)	1 (2.86)	1 (2.86)	1 (2.86)	7 (20.00)
Laparoscopy-group	43	1 (2.33)	0 (0.00)	0 (0.00)	1 (2.33)	0 (0.00)	0 (0.00)	2 (4.65)
*χ^2^*	-	-	-	-	-	-	-	4.453
*P*	-	-	-	-	-	-	-	0.035

## DISCUSSION

This study retrospectively analyzed records of adult patients who had undergone either laparoscopic or laparotomic cyst resection combined with Roux-en-Y hepaticojejunostomy to treat the Type-I congenital choledochal cysts. We found that both surgical methods were associated with similar operation times. However, the intraoperative blood loss was less and the time to recovery after the operation was shorter in the laparoscopy group compared to open surgery. Moreover, levels of SAMY, TBIL, and CRP in the laparoscopy group after the operation were better than those in the open group. Our results are similar to those reported by Altiti et al^7^, and they may have facilitated the rehabilitation of these patients.

A study by Zhuansun D et al,^8^ reported that laparoscopic operations take longer than open operations. However, this was not the case in our study, as we showed that the operation times were comparable between both groups. This difference may be due to the variability in surgeons’ experience and skill levels because of the high complexity of laparoscopic surgeries. The laparoscope inserted through a small abdominal puncture hole provides a wide field of vision due to its magnification function. In contrast, laparotomies require careful abdominal dissections (layer by layer) through a large abdominal incision, and the abdominal tissue is exposed to the air. In addition, during the operation, pulling, clamping, and other maneuvers cause tissue trauma leading to bleeding and long recovery times after the operations.^9^,^10^

At our hospital, we have successfully performed many laparoscopic cyst resections in combination with Roux-en-Y hepaticojejunostomy. Our surgeons, therefore, are highly skilled and have accumulated a rich experience. Thus, the operation times at our hospital are short and the operation times for both approaches are similar.^11^ In terms of the reduction of surgical trauma and the promotion of postoperative recovery, our results are similar to previous study.^12^

We found that the incidence of surgical complications in the laparoscopy group was significantly lower than that in the open group. Our results are in agreement with the results by Yeung F et al.^13^ When this operation is performed using a laparoscope, only puncture holes are needed, resulting in a clearer surgical field. This clear surgical field has been shown to lead to accurate procedures that reduce the amount of bleeding, the tissue trauma, and the risk of complications.^14^,^15^ However, due to the high difficulty level of the procedure, it is crucial to ensure that the operating surgeon is highly skilled in laparoscopic surgical techniques and is able to judge the timing of conversion from laparoscopic to open surgery if needed.

### Recommendations:

1. The surgeon should have experience in laparotomy and laparoscopy to ensure they can cope with the various emergencies that are possible during the operation.
2. When selecting the puncture holes, the patient’s body shape and cyst size should be considered, the number of holes for trocars should be varied according to the specific situation.
3. The cyst separation should be performed in a step wise manner, beginning with the front wall and the sides, then lifting the side wall and separating the rear wall.
4. When performing choledochojejunostomy, the intact serosa of the residual end of the common bile duct should be retained as much as possible, and ensuring that bile duct stenoses are not a problem and removing any residual stones is a priority before the anastomosis.
5. If a large cyst blocks the view of the laparoscope, affecting the operation space, and leading to uncontrollable bleeding, conversion to an open laparotomy should be considered.^16^

### Limitations:

This study was a single-center retrospective analysis with a small sample size, and short-term follow-ups. The included observation indicators are also few. Our conclusions are subjective and one-sided.

## CONCLUSION

Laparoscopic cyst resection combined with Roux-en-Y hepaticojejunostomy has the advantages of less trauma, faster recovery, less inflammation, and fewer complications than open surgery in adult patients with Type-I congenital choledochal cysts. However, due to the high difficulty level of the procedure, the operating surgeon should be highly skilled in laparoscopic surgical techniques and be able to judge the timing of conversion from laparoscopic to open surgery to confidently use this method in clinical practice.

### Authors’ contributions:

**HL**: Conceived and designed the study.

**HL** and **WH:** Collected the data and performed the analysis.

**HL:** Was involved in the writing of the manuscript and is responsible for the integrity of the study.

All authors have read and approved the final manuscript.
